# Danger Signals in the Initiation of the Inflammatory Response after Myocardial Infarction

**DOI:** 10.1155/2013/206039

**Published:** 2013-11-30

**Authors:** J. J. de Haan, M. B. Smeets, G. Pasterkamp, F. Arslan

**Affiliations:** Experimental Cardiology Laboratory, University Medical Center Utrecht, Heidelberglaan 100, Room G02.523, 3584 CX Utrecht, The Netherlands

## Abstract

During myocardial infarction, sterile inflammation occurs. The danger model is a solid theoretic framework that explains this inflammation as danger associated molecular patterns activate the immune system. The innate immune system can sense danger signals through different pathogen recognition receptors (PRR) such as toll-like receptors, nod-like receptors and receptors for advanced glycation endproducts. Activation of a PRR results in the production of cytokines and the recruitment of leukocytes to the site of injury. Due to tissue damage and necrosis of cardiac cells, danger signals such as extracellular matrix (ECM) breakdown products, mitochondrial DNA, heat shock proteins and high mobility box 1 are released. Matricellular proteins are non-structural proteins expressed in the ECM and are upregulated upon injury. Some members of the matricellular protein family (like tenascin-C, osteopontin, CCN1 and the galectins) have been implicated in the inflammatory and reparative responses following myocardial infarction and may function as danger signals. In a clinical setting, danger signals can function as prognostic and/or diagnostic biomarkers and for drug targeting. In this review we will provide an overview of the established knowledge on the role of danger signals in myocardial infarction and we will discuss areas of interest for future research.

## 1. Introduction

In 1994, Matzinger postulated a theory that the immune system may not be evolved to distinguish between self and non-self, but rather sense “danger” [1]. Danger signals, besides pathogen associated molecular patterns (PAMPs), can be intracellular molecules that are normally not exposed to the immune system, for example, cardiac myosin and mitochondrial DNA, but also proteins that are only upregulated during injury, such as heat shock proteins (HSP). Danger signals can therefore be divided into constitutive and inducible. Furthermore, danger signals can be classified as truly primal initiators, which do not require previous activation of antigen presenting cells (APC) or positive feedback signals, which can amplify or convert an ongoing inflammatory response [2]. This danger model explains the inflammatory response following myocardial infarction (MI), a situation where danger associated molecular patterns (DAMPs), and not pathogens, activate the immune system. For instance, extracellular matrix breakdown products released by the damaged myocardium and constituents of dying cardiomyocytes serve as danger signals in the infarcted myocardium, activating an inflammatory reaction. A certain amount of inflammation is required for proper healing and scar formation of the damaged myocardium. However, a prolonged presence of active leukocytes can be deleterious for the injured heart and can ultimately result in heart failure.

In the last decades we gained a lot of knowledge about danger signals, their receptors, and signaling pathways in different disease models. Also the inflammatory reaction in the heart is intensively studied and many DAMPs and their signaling pathways have been elucidated. Nevertheless, the precise actions of certain DAMPs in the heart remain unknown. In this review we will shortly address the concept of the danger model with its modulators and receptors. Subsequently, we summarize the current knowledge on danger signals after MI and discuss potential therapeutic possibilities and clinical applications.

## 2. Sensing Danger

**Figure 1:**
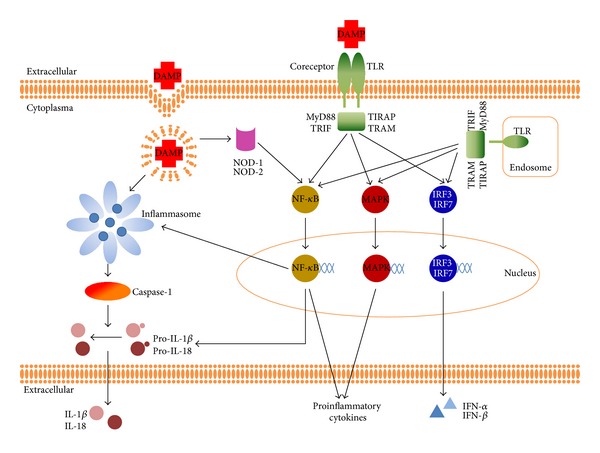
DAMP signaling through different PRRs. TLR activation by DAMPS triggers adaptor proteins MyD88, TRIF, TIRAP, or TRAM to activate various transcriptions factors. The subsequent translocation of NF-*κ*B and MAPK leads to the production of several proinflammatory cytokines. TRIF-dependent activation of transcription factors IRF3 and IRF7 results in the induction of type I interferon. Additionally, the TLR- NF-*κ*B pathway can induce the transcription of pro-IL-1*β*, pro-IL-18, and other components of the inflammasome pathway. Inflammasome activation is considered to depend on two distinct signals. The first signal via TLR and this might be the rate limiting step for inflammasome assembly and activity; the second signal via NLR which is responsible for inflammasome assembly, caspase-1 activation, and secretion of IL-1*β* and IL-18. Activation of NOD receptors results in activation of the NF-*κ*B pathway.

The primary mechanism by which the innate immune system can detect the presence of DAMPs is via pattern recognition receptors (PRRs). Ligands for these PRRs include molecules released by dying cells such as high mobility box 1 (HMGB1) and HSPs but also self-DNA and RNA and different extracellular matrix components. There are different classes of PRRs which sometimes share the same ligand and signaling pathways. In this review we will mainly focus on the membrane-bound Toll-Like Receptors (TLRs), the intracellular nucleotide binding and oligomerization domain (NOD)-like receptors (NLRs), and the Receptor for Advanced Glycation End-Products (RAGE). Both TLR and NLR can be activated through either PAMPs or DAMPs. Interaction with coreceptors like CD24-Siglec-G/-10 [3] or CD14/MD2 [4] allows the PRRs to discriminate between DAMPs and PAMPs and subsequently influence the level of inflammation [5]. In general, activation of PRRs results in the production of proinflammatory cytokines and recruitment and activation of immune cells (Figure 1).

### 2.1. Toll-Like Receptors

TLR is one of the best-described PRRs families. They are transmembrane receptors that can be divided into two groups, based on ligands and subcellular location. TLR1, TLR2, TLR4, TLR5, TLR6, TLR10, and TLR11 are located on the cell surface and scan the extracellular environment. TLR3, TLR7, TLR8, and TLR9 are present on the membrane of endosomal compartments of the cell and responsible for the recognition of, for example, microbial nucleic acids or self-DNA/RNA from dying cells.

TLR activation results in dimerization of the cytoplasmic signaling domains of TLRs. This subsequently initiates an intracellular signaling pathway involving specific adaptor molecules like MyD88 or TRIF. The MyD88 pathway can be used by all TLRs except TLR3 [6] and results in a cumulative activation of NF-*κ*B that mediates the transcription of proinflammatory cytokines. The TRIF pathway, independent of MyD88, can be activated via TLR3 and TLR4 [7] and results in the synthesis of interferon (IFN) (Figure 1).

The first article demonstrating an interaction between TLR and DAMPs was in 2000 by Ohashi who demonstrated that HSP60 could bind to and activate TLR4 [8]. Since then, the list of DAMPs that can activate TLRs is expanding rapidly. Depending on their biological background, TLRs can be activated by different types of DAMPs (Table 1).

In cardiac ischemic injury the role of TLRs has been intensively investigated [9, 10] and been linked to noninfectious tissue injury. TLR2 and TLR4 are the most extensively studied receptors in myocardial ischemic injury. TLR2 knockout (KO) mice have a reduced infarct size, improved cardiac function, and attenuated myocardial inflammation which is mediated via leucocytic TLR2 expression [11, 12]. Inhibition of TLR2 via an anti-TLR2 antibody also reduces infarct size and preserves cardiac function [13, 14]. In addition, TLR4 has a proinflammatory function during myocardial injury. TLR4 KO mice show reduced infarct sizes, attenuated adverse remodeling, and decreased inflammation [15, 16].

### 2.2. NOD-Like Receptors

NLRs are a class of intracellular receptors that recognize a variety of PAMPs and DAMPs and are highly conserved between species. So far, 22 different members have been identified in human, though the function of many remains unknown. All NLRs share the central nucleotide-binding and oligomerization (NACHT) domain, which is flanked by C-terminal leucine-rich repeats (LRRs) and N-terminal caspase recruitment (CARD) or pyrin (PYD) domains. Based on phylogenetic studies and similarities on domain structures, the NLR family can be divided into 3 subfamilies: the NODs, the NLRPs, and the IPAF.

The best known members of the NOD family are NOD1 and NOD2. Both initiate proinflammatory signaling via mitogen-activated protein kinase (MAPK) and NF-*κ*B pathways [17, 18]. There are many studies demonstrating a role for NOD1 and NOD2 in the recognition of peptidoglycan. However, there is still no evidence for direct binding to their ligands. In contrast to other NLRs, no endogenous ligands have been described for NOD1 and NOD2 so far.

Many of the IPAF and NLR subfamily members are well known for their capability to form large multiprotein complexes called inflammasomes that control caspase-1 activity. These include IPAF (or NLRC4) and NAIP (or NLRB1) from the IPAF subfamily and NLRP1, NLRP3, IPAF, and AIM2 from the NLRP family. Activation of the inflammasome involves a signaling complex consisting of a NLR protein, the adaptor ASC (apoptotic speck-containing protein with a CARD), and procaspase-1 and finally results in the formation of the pro-inflammatory cytokines IL-1*β* and IL-18 (Figure 1). For a more detailed description of inflammasome function, we refer to excellent review articles from Latz and Schroder [66, 67]. Notable, the important role of inflammasomes in myocardial ischemic injury has been described in several studies [68–70]. There are many different endogenous ligands that can activate inflammasomes (Table 1). For example, the AIM2 inflammasome can sense cytoplasmic DNA [56, 71] and the NLRP3 inflammasome can be activated via C3a [72], extracellular acidosis [73], and extracellular Ca^2+^ [74].

Although they have not been studied as extensively as TLR, there are a number of studies that demonstrate a role for NLR in myocardial ischemic injury. Already in 2001, it was demonstrated that caspase-1 inhibition reduces myocardial ischemia injury [75], whereas activation of NOD1 induces cardiac dysfunction and modulates cardiac fibrosis and cardiomyocyte apoptosis [76]. More recently, studies with KO mice demonstrate the direct role of NLR in myocardial ischemic injury. NLRP3 KO mice show improved cardiac function and decreased infarct size [69]. Similar results are found using either ASC or caspase-1 KO mice [68].

### 2.3. Receptor for Advanced Glycation End-Products

RAGE is the only AGE receptor that has been implied to play a role in DAMP recognition. It is a membrane bound multiligand receptor that can recognize, besides AGE, multiple ligands including HMGB1, amphoterin, and several S100 proteins [77, 78]. Recently, several secreted isoforms of RAGE have been described that lack the transmembrane domain and the cytosolic tail which might act as a “decoy” receptor [79–81].

RAGE signaling appears to be detrimental after MI, since recombinant HMGB1 or recombinant S100A8/A9 worsened ischemia-/reperfusion injury. Furthermore, RAGE KO mice show reduced tissue damage and less inflammation after MI [40, 82].

### 2.4. Synergy and Cross-Talk

There is a high level of interplay between the different PRRs family members and they also share several common ligands like HMGB1, S100A8/A9 complex, and *β*-sheet fibrils [40, 41, 53, 54, 83]. It is generally accepted that IL-1*β* release by the inflammasome requires two distinct signals where the first signal primes the cell via TLR. As most cells do not constitutively express high amounts of pro-IL-1*β*, TLR activation and subsequent NF-*κ*B translocation to the nucleus results in increased expression of pro-IL-1*β*, pro-IL-18, and other inflammatory components like NLRP3 [69, 84–86]; the secondary (endogenous) stimulus then promotes inflammasome assembly, activation, and subsequent secretion of IL-1*β* and IL-18. The necessity of costimulation via two receptor types might function as a fail-safe mechanism to make sure that only in the presence of a real stimulus such as tissue injury, the activation of the proinflammatory pathways occurs.

Another example of the interaction between different PRRs family members is demonstrated after costimulation of both TLR2 and NOD1 [87] which result in enhanced proliferation, expansion, and effector function of T cells. In contrast, costimulation with TLR2 and NOD2 is responsible for an augmented inflammatory response [88]. Interestingly, there can also be a negative regulation when TLR2 and NOD2 are simultaneously activated, NOD2 has also been described to play a suppressive function in TLR2 signaling [89].

There is also evidence that endogenous ligands can interact with each other to enhance or dampen the inflammatory response that they elicit. A classic example is HMGB1 that was first identified as a DAMP. However, several studies demonstrated recently that the formation of complexes with other proinflammatory ligands results in enhanced inflammation instead of HMGB1 alone [90]. For example, HMGB1 can facilitate the transfer of LPS to CD14 [91] and enhances nucleosome binding to TLR2 [61] and dsDNA binding to TLR9 [92].

## 3. High Mobility Box 1

High mobility box 1 (HMGB1, also known as HMG1, amphoterin, or p30) was discovered as a nonhistone DNA binding protein, involved in stabilization of DNA and promotion of gene transcription. Recent discoveries established the inflammatory role of HMGB1. Scaffidi et al. demonstrated that necrotic cells release HMGB1 and hereby elicit inflammation. On the other hand during apoptosis HMGB1 is firmly attached to the chromatin, thus preventing its release and subsequent immune responses [34]. HMGB1 exhibit specific danger signal functions, because it is only released by damaged cells and activates immune responses.

HMGB1 signals through RAGE, TLR2, and TLR4, thereby stimulating macrophages, monocytes, and neutrophils to secrete the proinflammatory cytokines TNF-*α*, IL-1, IL-6, IL-8, and macrophage inflammatory protein (MIP) [31–34]. Furthermore, HMGB1 induces the expression of adhesion molecules, for example, intercellular adhesion molecule 1 (ICAM-1) and vascular adhesion molecule 1 (VCAM-1) on endothelial cells. HMGB1 has been studied as an inflammatory mediator in a range of diseases, such as ischemia in the liver [93] and brain [94]. Also after MI there is an immediate increase of plasma HMGB1 levels in rat and human [95, 96]. In the infarcted myocardium of rodents the expression of HMGB1 is upregulated after 2 [82] or 3 days [95] depending on the model used. Furthermore, the elevated levels of HMGB1 in patients with acute coronary syndrome [95, 96] are associated with a decreased heart rate recovery, a marker of autonomic function defined as the fall in heart rate during the first minutes of exercise [97], and adverse LV remodeling [98] and predict secondary events, such as pump failure and cardiac rupture [95]. This might reflect that increased amounts of HMGB1 are detrimental. Surprisingly, injection of HMGB1 in rat hearts after permanent coronary ligation improved cardiac function by modulating inflammation via reducing the accumulation of dendritic cells [99] and HMGB1 delivered to the heart by a hydrogel induced vascularization and improved cardiac function [100]. Furthermore, treatment of anti-HMGB1 showed enhanced adverse LV remodeling, although it prevented the upregulation of the cytokines TNF*α* and IL-1*β* and the influx of macrophages [95]. In addition, in a mouse model of permanent coronary ligation, local injection of exogenous HMGB1 improved myocardial function [101] and in transgenic mice overexpressing HMGB1 survival and cardiac function was improved after MI [102]. However, there are also studies showing opposite effects. For example, Andrassy et al. demonstrated that systemic injection of an antagonist of HMGB1 improves cardiac function after ischemia-reperfusion in WT mice and recombinant HMGB1 worsened cardiac function. Both the antagonist or the recombinant protein had no effect on RACE KO mice, which suggest that HMGB1 signaling through RAGE inhibits the reparative response after MI. Also, administration of ethyl pyruvate, which inhibits the release of HMGB1, preserves cardiac function after extended myocardial ischemia followed by reperfusion [103]. Interestingly, preconditioning with HMGB1 shows protection against ischemia-reperfusion injury [104].

These contradictory results can partly be explained by the different models that are used. In permanent coronary ligation models angiogenesis is a prominent mediator of cardiac remodeling and improves cardiac repair. HMGB1 appears to have beneficial effects in this model, which can be assigned to the role of HMGB1 in angiogenesis [102]. The detrimental effects of HMGB1 are observed in ischemia-reperfusion models, in which inflammation plays a great role and might be aggravated by HMGB1. However, the amount of leukocytes is comparable in early time points after permanent ligation or reperfusion injury [105]. Thus in some cases the improved cardiac function in the different models may be explained by the route and time point of administration. Local injection after MI with HMGB1 improved cardiac function and in contrast systemic HMGB1 just before MI worsened cardiac function. Furthermore, low dose HMGB1 seems to be beneficial and high dose of HMGB1 to be harmful [106]. Apart from the great knowledge we already have about the role of HMGB1 as a danger signal, further research is required to solve the disagreement whether HMGB1 is deleterious or beneficial in ischemic heart diseases and how this can be implemented in the clinic. It should be taken into account that ischemia-reperfusion models are more clinically relevant, since all patients undergo reperfusion therapy (either pharmacologically or mechanically) in the setting of acute MI.

## 4. Heat Shock Protein 60 and 70

HSPs gain their name because their expression is upregulated as a result of high temperatures. Later it became clear that various kinds of stress responses can enhance the expression and release of HSPs. During homeostasis HSPs are expressed by numerous cell types and function as chaperones in protein folding and translocation; however, upon injury HSPs can function as danger signals. For instance, in rats, HSP27, HSP72, and HSP60 were significantly induced following coronary artery ligation, whereby the expression of HSP60 was correlated with the development of heart failure [107, 108]. In human, HSP27 and HSP60 expressions are increased in the myocardium of patients with ischemic cardiomyopathy [109] and circulating HSP70 levels are increased after acute MI [38, 110, 111]. HSP60 and HSP70 are widely studied as danger signals after MI. Endogenous and exogenous HSP60 signals via TLR4-MyD88-p38/NF-*κ*B in cardiomyocytes and augments pro-inflammatory cytokine production, such as IL-1*β*, TNF-*α*, and IL-6 [36, 37]. Furthermore, in patients with acute coronary syndrome pro-inflammatory HSP60-reactive CD4^+^CD28^null^ T cells are found [112], which indicates that these T cells are activated by HSP60 stimulated APC. Likewise, this suggests that HSP60 functions as a primal danger signal after acute coronary syndrome. Similarly, HSP70 is elevated after MI and related to inflammation and TLR4 signaling [38, 111]. Moreover, HSP70 can activate monocytes through CD14, which results in a release of pro-inflammatory cytokines IL-1*β*, TNF-*α*, and IL-6 [113]. Surprisingly, rats administered with bimoclomol, which increases HSP70 levels, exhibit decreased infarct size after coronary ligation. However, bimoclomol was given before MI induction, which is not a good clinically relevant model [114]. More studies are necessary to establish the exact role of HSP70 as a danger signal and how this can be used in the clinic.

In summary, HSP60 and HSP70 are both upregulated after MI. HSP60 has a well-established role as a danger signal, while HSP70 has only been associated with inflammation. More studies are warranted to define the role of HSPs in the clinical setting.

## 5. Mitochondrial DNA

It is known that bacterial DNA has robust immune properties. The CpG sequence abundantly present on prokaryotic DNA serves as a PAMP and activates B cells, macrophages, and DCs through the intracellular TLR9 [115]. Mitochondrial mtDNA, originating from bacteria, contains the same CpG sequence and can thereby function as a DAMP. Zhang et al. show that traumas, for example, myocyte injury, trigger the release of mtDNA and that circulating mtDNA provokes inflammation in polymorphonuclear neutrophils [116]. Interestingly, Oka et al. show that mtDNA can also autonomously activate TLR9 by escaping from autophagy-mediated degradation and in this way aggravate pressure-overloaded heart failure [117]. As a response to this paper, Konstantinidis and Kitsis postulate that this pathway may be of greater importance after MI, because inflammation is more pronounced in MI compared to chronic heart failure [118]. In addition, patients suffering from MI show increased levels of circulating mtDNA [119]. Despite the lack of studies on the effects of mtDNA in ischemic heart diseases, it can be speculated that mtDNA, released by necrotic cells or escaped from autophagy, serves as a danger signal after MI.

## 6. Fibronectin-EDA

Fibronectin (FN) is a dimeric glycoprotein found in the ECM. Different isoforms exist due to alternative splicing. The FN-EDA splice variant is highly expressed during embryogenesis and upregulated upon injury. FN-EDA can bind the integrins *α*9*β*1 and *α*4*β*1, thereby mediating cell adhesion [120]. Furthermore, FN-EDA can activate leukocytes through TLR2 and TLR4 in vitro [28, 29]. FN-EDA is upregulated after MI in mice [121] and human (unpublished data). Moreover, EDA KO mice show less inflammation, reduced monocyte recruitment, and improved cardiac function after MI [121]. In addition, in ischemic stroke, constitutive expression of FN-EDA significantly increased neutrophil and macrophage infiltration, inflammatory cytokines, and brain injury. Interestingly, treatment with a specific TLR4 inhibitor abolished these effects, which suggests that FN-EDA by signaling through TLR4 promotes inflammation and subsequent injury [30]. Although some evidence is still lacking, it can be speculated that EDA functions as an inducible danger signal after MI by attracting and activating leukocytes through TLR and/or integrin signaling.

## 7. Matricellular Proteins as Danger Signals

Matricellular proteins are nonstructural proteins expressed in the ECM and are upregulated upon injury. Many matricellular proteins are shown to be upregulated after MI and play an important role in the reparative response. An excellent review has been published about the role of matricellular proteins in the infarcted myocardium [122]. Some matricellular proteins also show characteristics of an inducible danger signal and those will be discussed here.

### 7.1. Tenascin-C

Tenascin-C (TN-C) is a glycoprotein mostly expressed in the ECM during development [122] and is normally not abundantly expressed in adult tissue. TN-C is upregulated under pathological conditions, such as pulmonary fibrosis [123] and MI [124–127] and is closely associated with inflammation [123]. Despite the fact that TN-C is upregulated in inflammatory diseases, not much is known yet about its role in vivo and whether TN-C functions as a primal danger signal. In vitro, TN-C supports lymphocyte tethering and rolling under flow conditions [128, 129], and soluble TN-C has also been shown to inhibit T cell activation and proliferation [129, 130] through the *α*5*β*1 integrin [131]. In a model of rheumatoid arthritis, TN-C shows to signal through TLR4, thereby increasing inflammation [43]. Moreover, human macrophages secrete more of the proinflammatory cytokines IL-6, IL-8, and TNF upon TN-C stimulation through TLR4 [43].

TN-C KO mice show no cardiac dysfunction in the absence of injury, suggesting that TN-C does not play a significant role in homeostasis of the heart. However, TN-C KO mice show less fibrosis and remodeling after MI. Unfortunately, in this study the inflammatory actions of TN-C were not studied. Future research should focus on the immune modulatory actions of TN-C in the infarcted myocardium to investigate whether TN-C might be an interesting candidate in controlling the inflammatory response.

### 7.2. Osteopontin

Osteopontin (OPN or Eta-1) was originally identified as a bone matrix protein. Later it became clear that OPN is also a cytokine, secreted by many immune cells. OPN is constitutively expressed by macrophages [132] and is upregulated in numerous cells types upon injury [133]. In macrophages OPN has been shown to function in the migration [134], activation [135], phagocytosis [133], and inflammatory cytokine production [133, 136]. Furthermore, OPN acts as a chemoattractant for neutrophils and DCs [136, 137]. Interestingly, OPN can activate DCs to produce IL-12 and TNF-*α*, which suggest that OPN functions as a primal danger signal. Additionally, OPN activated DCs stimulate a Th1 response when cocultured with naïve T cells [138–140].

OPN is upregulated in experimental models of infarction in mice [141, 142], rats [143], dogs [142], pigs [144], and in human patients suffering an acute MI [132, 145]. OPN KO mice show excessive dilation and reduced collagen deposition of the LV upon MI [141]. Unfortunately, the mechanisms behind the decreased collagen deposition and the role of inflammatory cells are not studied, so whether OPN functions as a danger signal in MI cannot yet be defined. In patients, OPN levels are increased after MI [145] and are predictive for long-term outcome. Furthermore, their role as an immune modulator has been established in many other diseases [133, 137, 146], so it can be speculated that OPN functions as a danger signal. However, more research is required to unravel the role and function of OPN in cardiac ischemic injury and subsequent repair, as this might lead to new therapeutic options.

### 7.3. CCN1

The CCN family obtained its name from the first members described, cysteine-rich protein 61(CYR61), connective tissue growth factor and nephroblastoma overexpressed protein. CCN are considered as matricellular proteins and have been shown to be involved in many cellular processes such as adhesion, migration, and proliferation, mainly via modulating signaling of other molecules [122]. CCN1, also known as CYR61, is highly upregulated in the infarcted myocardium in mice [147, 148] and human [148]. Furthermore, CCN2 and CCN4 are upregulated after MI, but little is known about their inflammatory actions. Interestingly, CCN1 can activate proinflammatory genes in macrophages by binding to *α*_M_ *β*_2_ and syndecan-4 [149]. However, CCN1 inhibits the migration of macrophages and lymphocytes in autoimmune myocarditis [150]. To explain the paradoxical role of CCN1, Löbel and colleagues showed a diphasic immune modulator response for CCN1; initial stimulation with CCN1 attracts and activates leukocytes; however, prolonged CCN1 stimulation and enhanced secretion of CCN1 by leukocytes immobilize systemic leukocytes [151]. It can be speculated that CCN1 may function as a danger signal after MI by attracting and activating leukocytes, however, in vivo studies are necessary to state this.

### 7.4. Galectins

Galectins are a family of proteins that have an affinity for binding *β*-galactosides sugars. So far, 15 different galectins have been described. Some galectins have been characterized as matricellular proteins [152], including galectin-1 and galectin-3. Both galectins have been shown to function as a DAMP [153] and found to be upregulated after MI in human [154, 155] and mice [155]. Galectin-3 can support neutrophil adhesion, migration, and activation. Furthermore, galectin-3 functions as a chemoattractant for macrophages [156] and both galectin-1 and galectin-3 can alternatively activate macrophages. Importantly, galectin-1 is able to augment DC migration, and induce maturation [157]. Interestingly, Seropian and colleagues recently showed that galectin-1 prevents cardiac inflammation in a mouse model of acute MI [155]. These studies might suggest that different galectins have distinct functions in inflammation. Not many in vivo studies have been conducted yet to establish the role of galectins as a DAMP following MI. However, it can be hypothesized that both galectin-1 and galectin-3 play a role in the inflammatory reaction.

## 8. Clinical Implications

In the clinical context, understanding the role of danger signals could have important applications. Hypothetically, all danger signals that can be measured in blood may serve as biomarkers for diagnostic and/or prognostic purposes. For example, high plasma levels of HMGB1 are shown to be strongly associated with increased mortality in patients with STEMI independent of age, sex, troponin I, and CK-MB [158]. In addition, in patients with unstable angina or NSTEMI, high serum levels of HBGB1 are associated with higher mortality during 49 month followup [159]. Both of these studies demonstrate that HMGB1 levels can be used as a new prognostic biomarker in patients with acute coronary syndrome.

Also HSP70 might be a new biomarker for patients with heart failure. HSP70 is elevated in AMI patients and after 14 days HSP70 levels were higher in patients with heart failure compared to patients without heart failure [38]. In addition, Li et al. showed that elevated levels of HSP70 correlate with the progression of HF [110].

In critically ill patients, high levels of FN-EDA correlate with increased risk for progression to acute hypoxemic respiratory failure [160]. It would be interesting to study the prognostic value of FN-EDA levels in patients with acute coronary syndrome.

On a theoretical basis, danger signals are excellent therapeutic targets because they are only released or upregulated after injury. Nevertheless, DAMP-induced inflammation is also essential for proper healing of the infarcted area. Hence, it is of utmost importance to establish the exact time frame in which intervention is optimal. Injection of HMGB1 or an antibody against HMGB1 has extensively been studied in animal models. However, no uniform effects were observed. In some models HMGB1 seems to prevent cardiac remodeling, however, in other models HMGB1 appears to be detrimental. This is probably due to the two different MI models used to study cardiac remodeling: the permanent coronary ligation and ischemia-reperfusion. Before HMGB1 or anti-HMGB1 can be brought to the clinic, it is crucial that the mechanism of action and the therapeutic window are established. In addition to targeting a DAMP for therapeutic intervention, it is also an option to target receptors. It is challenging to inhibit a certain receptor in order to prevent DAMP-PRRs interaction, since the same PRRs are necessary for host defense. Nevertheless, a few examples can be given. An anti-TLR2 antibody reduced leukocyte influx and infarct size after MI in both mice and pigs [13]. Furthermore in a brain ischemic-reperfusion model a TLR4 inhibitor reduced injury [30] and because many DAMPs signal through TLR4 this is also an interesting candidate for the treatment of MI.

## 9. Concluding Remarks

**Figure 2:**
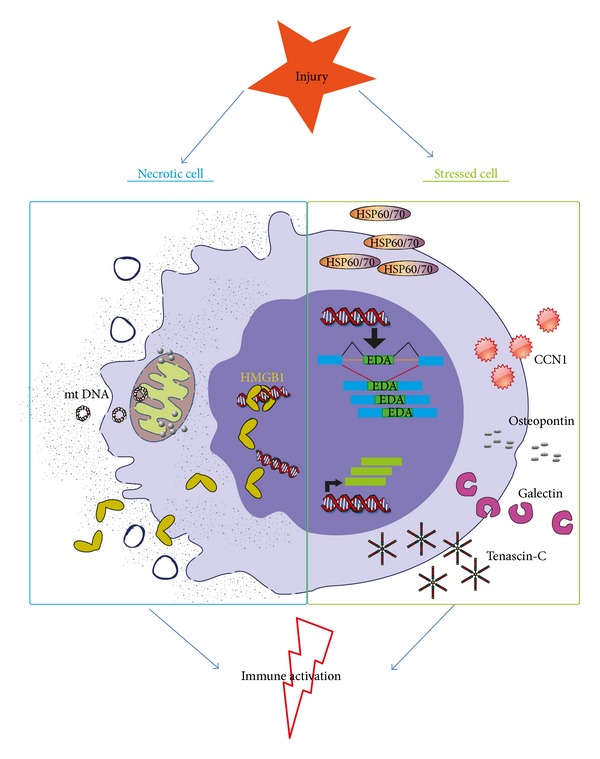
Proposed simplified mediators of danger signal release during myocardial infarction. Necrotic cells in the myocardium are leaky and release a subset of DAMPs, for instance, mtDNA and the DNA binding protein HMGB1. Furthermore, viable cells get stress signals from their surroundings and start to produce and secrete a range of proteins. These cells start the production of the EDA splice variant of fibronectin, HSPs and the matricellular proteins CCN1, osteopontin, galectins, and tenascin-C. Both the proteins released by necrotic cells and the produced proteins by stressed cells are able to activate or aggravate the immune response in the heart following MI.

The danger model has shown to be useful as a theoretical framework in cardiovascular science. Interesting new DAMPs are identified that might influence the deleterious and beneficial effects of the immune system in tissue healing and scar formation.  Figure 2 shows how and which danger signals can be released following MI. However, for only a few danger signals a true causal relationship has been established in MI and for many danger signals research is still ongoing to establish their effects in MI. It will be interesting to use conditional KO and bone marrow chimera approaches to investigate which cells release and produce the danger signal of interest. Furthermore, ECM breakdown products and matricellular proteins are of main interest to study as potential danger signals. Danger signals, or DAMPs, may also be used as diagnostic and prognostic markers. Additional studies on correlation between specific danger signals and primary and/or secondary outcome are necessary before clinical application. Intracellular and inducible DAMPs, such as mtDNA and matricellular proteins, are interesting candidates for therapeutic interventions, considering that they are only present in the injured environment.

To conclude, extended research is necessary to define the role of specific danger signals in MI. Regardless, DAMPs may be of additive value in the clinic as diagnostic/prognostic markers and therapeutic targets.

## Figures and Tables

**Table 1:** DAMPs and their receptors.

	Endogenous ligand	TLR	NLR	Others	References
Proteins, peptides	Amyloid-*β*	TRL2, TLR4/6	NLRP3		[19–21]
	Complement membrane attack complex		NLRP3		[22]
	*α* and *β* defensins	TLR4	NLRP3		[23, 24]
	Eosinophil-derived neurotoxin	TLR2			[25]
	Fetuin A	TLR4			[26]
	Fibrinogen	TLR4			[27]
	Fibronectin-EDA	TLR2, TLR4			[11, 28–30]
	HMGB1	TLR2, TLR4, and TLR9		RAGE	[31–34]
	HSP60	TLR2, TLR4			[35–37]
	HSP70	TRL2, TLR4/6			[38]
	Osteopontin	TLR9 (MyD88)			[39]
	S100A8/A9	TLR4	NLRP3	RAGE	[40–42]
	Tenascin-C	TLR4			[43]
	TNF-*α*	NLRP3			[44]

Proteoglycans, Glycosaminoglycans	Biglycan	TLR2, TLR4	NLRP3		[45, 46]
	Hyaluronic acid fragments	TLR2, TLR4			[47, 48]
	Versican	TLR2/6			[49]

Fatty acids, lipoproteins	Cholesterol crystals		NLRP3		[50]
	Oxidized LDL	TLR2,TLR4, and TLR4/6			[20, 51]
	Saturated fatty acids	TLR4			[52]
	Serum amyloid A	TLR2, TLR4	NLRP3		[53–55]

Nucleic acids	Mitochondrial DNA	TLR9	AIM2, NLRP3		[54, 56, 57]
	mRNA	TLR3			[58]
	ss RNA	TLR7, TLR8			[59]

Protein-nucleotide complexes	IgG-chromatin complexes	TLR9			[60]
	HMGB1-nucleosome complex	TLR2			[61]

Purine metabolites	ATP		NLRP1b, NLRP3		[62, 63]
	Uric acid	TLR2, TLR4	NLRP3		[64, 65]
